# Isocaloric substitution of carbohydrates with protein: the association with weight change and mortality among patients with type 2 diabetes

**DOI:** 10.1186/s12933-015-0202-7

**Published:** 2015-04-18

**Authors:** Marjo JE Campmans-Kuijpers, Ivonne Sluijs, Ute Nöthlings, Heinz Freisling, Kim Overvad, Elisabete Weiderpass, Guy Fagherazzi, Tilman Kühn, Verena A Katzke, Amalia Mattiello, Emily Sonestedt, Giovanna Masala, Claudia Agnoli, Rosario Tumino, Annemieke MW Spijkerman, Aurelio Barricarte, Fulvio Ricceri, Saioa Chamosa, Ingegerd Johansson, Anna Winkvist, Anne Tjønneland, Diewertje Sluik, Heiner Boeing, Joline WJ Beulens

**Affiliations:** Julius Center for Health Sciences and Primary Care, University Medical Center Utrecht, STR 6.131, P.O. Box 85500, 3508 GA Utrecht, The Netherlands; Department of Nutrition and Food Sciences, University of Bonn, Bonn, Germany; International Agency for Research on Cancer (IARC-WHO), Lyon, France; Department of Public Health, Section for Epidemiology, Aarhus University, Aarhus, Denmark; Department of Community Medicine, Faculty of Health Sciences, University of Tromsø, The Arctic University of Norway, Tromsø, Norway; Institut Gustave-Roussy, Villejuif, France; Division of Cancer Epidemiology, German Cancer Research Center (DKFZ), Heidelberg, Germany; Dipartimento Di Medicina Clinica E Chirurgia Federico II University, Naples, Italy; Department of Clinical Sciences Malmö, Lund University, Malmö, Sweden; Molecular and Nutritional Epidemiology Unit, Cancer Research and Prevention Institute – ISPO, Florence, Italy; Epidemiology and Prevention Unit, Fondazione IRCCS Istituto Nazionale dei Tumori, Via Venezian 1, 20133 Milano, Italy; Cancer Registry and Histopathology Unit, “Civic - M.P.Arezzo” Hospital, ASP, Ragusa, Italy; National Institute for Public Health and the Environment, Centre for Nutrition, Prevention and Health Services, PO Box 1, 3720 BA Bilthoven, The Netherlands; Jefe Del Servicio De Epidemiologia, Prevencion Y Promocion De La Salud, Instituto de Salud Pública, Leyre 15, 31003 Pamplona, Spain; Unit of Cancer Epidemiology - CERMS, Department of Medical Sciences, University of Turin and Città della Salute e della Scienza Hospital, Turin, Italy; Public Health Division of Gipuzkoa, BioDonostia Research Institute, Health Department of Basque Region, San Sebastian, Spain; Institutionen för odontologi, Umeå university, Umeå, Sweden; Institutionen för medicin, Göteborgs universitet, Göteborg, Sweden; Diet, Genes and Environment, Danish Cancer Society Research Center, Copenhagen, Denmark; Division of human nutrition, WU Agrotechnology & Food Sciences, P.O. Box 8129, 6700 EV Wageningen, The Netherlands; Department of Epidemiology, German Institute of Human Nutrition, Potsdam-Rehbrücke, Germany

**Keywords:** Carbohydrates, Protein, Type 2 diabetes, Body weight (change), Mortality risk

## Abstract

**Background:**

The health impact of dietary replacement of carbohydrates with protein for patients with type 2 diabetes is still debated. This study aimed to investigate the association between dietary substitution of carbohydrates with (animal and plant) protein and 5-year weight change, and all-cause and cardiovascular (CVD) mortality risk in patients with type 2 diabetes.

**Methods:**

The study included 6,107 diabetes patients from 15 European cohorts. Patients with type 1 diabetes were excluded. At recruitment, validated country-specific food-frequency questionnaires were used to estimate dietary intake. Multivariable adjusted linear regression was used to examine the associations between dietary carbohydrate substitution with protein and 5-year weight change, and Cox regression to estimate hazard ratios (HRs) for (CVD) mortality.

**Results:**

Annual weight loss of patients with type 2 diabetes was 0.17 (SD 1.24) kg. After a mean follow-up of 9.2 (SD 2.3)y, 787 (13%) participants had died, of which 266 (4%) deaths were due to CVD. Substitution of 10 gram dietary carbohydrate with total (ß = 187 [75;299]g) and animal (ß = 196 [137;254]g) protein was associated with mean 5-year weight gain. Substitution for plant protein was not significantly associated with weight change (β = 82 [−421;584]g). Substitution with plant protein was associated with lower all-cause mortality risk (HR = 0.79 [0.64;0.97]), whereas substitution with total or animal protein was not associated with (CVD) mortality risk.

**Conclusions:**

In diabetes patients, substitution with plant protein was beneficial with respect to weight change and all-cause mortality as opposed to substitution with animal protein. Therefore, future research is needed whether dietary guidelines should not actively promote substitution of carbohydrates by total protein, but rather focus on substitution of carbohydrates with plant protein.

## Background

Overweight is very common among patients with type 2 diabetes, and weight loss may reduce mortality by 25% in these patients [1]. A low-fat (and thus relatively high carbohydrate) diet has been recommended to patients with type 2 diabetes to prevent cardiovascular diseases and as a means to lose weight [2-5], although initially, carbohydrates were avoided due to their postprandial glucose and insulin raising effects [6]. However, there is still debate on the optimal percentage of carbohydrates and its substitutions in the calorie reduced diet for patients with type 2 diabetes [7].

Recent studies suggested that high dietary protein intake contributes to weight loss due to higher satiety with a high-protein diet [8]. Recently, an ad-libitum intervention study in healthy obese participants showed a modest increase in dietary protein to be beneficial for the maintenance of weight loss after 26 weeks [9], whereas an observational study in healthy people showed that a higher protein intake was associated with weight gain, mostly as fat mass after 6 years [10]. Iso-energetic studies found no significant difference on weight change between high-protein or high-carbohydrate diets in the short term [8,11] and inconclusive evidence in the long term [12,13]. Moreover, one study suggested differences in effects of animal and plant protein, with a direct association of animal protein and risk of overweight and obesity, and an inverse association for plant protein [14].

Despite these potential effects on body weight, the evidence for a relationship between total protein intake and all-cause mortality risk or cardiovascular diseases is inconclusive in the general population [12]. There is suggestive evidence for an association between long-term low-carbohydrate–high-protein diets and a higher all-cause mortality risk and for an inverse association between plant protein and cardiovascular (CVD) mortality [12].

We are aware of only three studies in patients with type 2 diabetes. Two short term randomized trials found no significant difference in weight change after one year of a high protein diet compared to a regular diet [15,16]. After twelve weeks, women with type 2 diabetes on a high-protein diet achieved significantly higher total and abdominal fat loss compared with women on a low-protein diet, whereas no differences were found in men [17]. We are not aware of any long term studies in patients with type 2 diabetes on the relationship between protein intake and either weight change or (CVD) mortality.

The aim of this study was to investigate the association between dietary carbohydrate intake and substitution with (animal and plant) protein and subsequent weight (and waist) change in patients with type 2 diabetes. Second, we aimed to investigate prospectively the associations with risk of CVD and all-cause mortality.

## Methods

### Study population

Within the European Prospective Investigation into Cancer and Nutrition (EPIC) [18], a subcohort was defined of participants with a confirmed diagnosis of diabetes mellitus at recruitment as described earlier [19]. The following EPIC-centers have contributed to this project: Aarhus and Copenhagen (Denmark), Heidelberg and Potsdam (Germany), Florence, Naples, Ragusa, Turin, and Varese (Italy), Bilthoven and Utrecht (the Netherlands), Navarra and San Sebastian (Spain), and Malmö and Umeå (Sweden). Self-reports of diabetes at recruitment were confirmed by a second source of information, i.e. contact to a medical specialist or practitioner, self-reported use of medication for diabetes treatment, repeated self-report of diagnosis during follow-up or record linkage to a diabetes registry or a glycated hemoglobin (HbA1c) level above 42 mmol/mol (6%). This cohort study was conducted according to the Declaration of Helsinki and was approved by a local ethical review committee of each center and of the International Agency for Research on Cancer in Lyon, France. All subjects provided written informed consent.

Of 7,048 initial self-reports, 5,542 diabetes diagnoses were confirmed. As a result of verification efforts in other projects within the EPIC, a further 870 prevalent diabetes cases without self-reported diabetes at recruitment were identified. This led to a subcohort comprising 6,412 individuals with confirmed diabetes at recruitment [20]. We excluded participants with missing dietary information (N = 42), participants in the highest or lowest 1% of the ratio of total energy intake/estimated energy requirement (N = 177), and one deceased participant with missing date of death, and 85 patients with type 1 diabetes, resulting in 6,107 participants (3,328 men and 2,779 women).

For the analytical sample on weight change, participants with missing weight or extreme anthropometry at recruitment or follow-up [height <130 cm, BMI < 16 kg/m^2^, waist circumference <40 or >160 cm and waist circumference < 60 cm with BMI > 25 kg/m^2^] were excluded on top of the above mentioned exclusions. Furthermore, participants with weight change >5 kg/year (N = 2) or those without follow-up data on weight or BMI (N = 2,067), this included the cohorts of Turin and Ragusa and parts of the cohort in Naples (all in Italy) were excluded. This analytical sample included 4,082 participants (2,255 men and 1,827 women). For 1,878 participants waist circumference change could be analyzed.

For cardiovascular and all-cause mortality, the analysis sample consisted of the entire population of 6,107 participants.

### Dietary assessment

In EPIC, dietary intake during the previous year was assessed at recruitment by means of country-specific validated dietary questionnaires [18], either quantitative dietary questionnaires with individual portion sizes (in France, Spain, the Netherlands, Germany and Italy, except Naples) or semi-quantitative food frequency questionnaires (in Denmark, Naples (Italy), and Sweden), that were developed and validated locally [18]. Correlation coefficients for the relative validity measured with food frequency questionnaires (FFQ) varied for carbohydrates from 0.46 to 0.76 in women and from 0.40 to 0.84 in men; for protein from 0.26 to 0.67 in women and from 0.35 to 0.71 in men [21].

Basal metabolic rate (BMR) was estimated using the Schofield equations [22]. Participants with a ratio of energy intake to BMR <1.14 were defined as energy under-reporters, whereas those with a ratio >2.40 were classified as energy over-reporters according to the Goldberg cut-offs [23].

Glycemic Index (GI) values of foods were obtained in a standardized manner [24] from the Foster-Powel table [25], British values [26] and internet updates http://www.glycemicindex.com using glucose as the reference. GI values were updated in 2009, using the recently published table by Atkinson et al. [27].

### Assessment of anthropometric measures and weight change

At recruitment, body weight [kg] and height [cm] were measured without shoes according to standardized procedures [28]. Waist circumference [cm] was measured either at the midpoint between the lower ribs and iliac crest or at the narrowest torso circumference. Weight and waist circumference measurements were corrected to account for protocol differences between centers as previously described [28]. For normally dressed participants without shoes 1.5 kg for weight and 2.0 cm for waist circumference were subtracted from the original measurement; for participants in light clothing without shoes 1 kg was subtracted from the weight. BMI was calculated as body weight [kg] divided by height squared [m^2^].

At follow-up, weight and waist circumferences [cm] were self-reported in all centers. Weight change [g/y] was calculated by subtracting the weight at recruitment from the follow-up weight, subsequently dividing this by the number of years of follow-up. For the 5-year weight change [g/5y] this result was multiplied by 5. The same applies for the calculation of 5-year waist circumference change [cm/5y].

### Measurements of non-dietary factors

At recruitment, lifestyle- and health related variables were collected using a general questionnaire. Physical activity was indexed into four categories (inactive, moderately inactive, moderately active and active) based on the validated Cambridge Physical Activity Index [29,30]. Information on smoking status was coded into three categories (never, former, current) and smoking intensity was assessed in eight categories (never; former smokers divided in three categories: time since quitting ≤10 years, time since quitting between 11–20 years, time since quitting > 20 years; current smokers, also divided three categories: smoking 1–15, 16–25, and over 25 cigarettes a day, and one category with current pipe, cigar or occasional smokers). Diabetes duration was calculated from the date of the confirmed diagnosis as mentioned above or by self-reported age at diagnosis. Insulin use was defined by self-reported diabetes related medication at recruitment. Education was assessed in four categories: primary education, technical school, secondary school and university degree.

### All-cause and cardiovascular mortality

Information on vital status, cause and date of death, were obtained by using follow-up mailings and subsequent inquiries to municipal registries, regional health departments, physicians, or by record linkages with local, regional, or central cancer registries, boards of health, or hospitals (Germany), or death indexes (other countries). Mortality data were coded according to the International Classification of Diseases (ICD-10). Primary and secondary causes of death were combined for CVD mortality (ICD-10 [I00-I99]).

### Statistical analysis

Baseline characteristics are presented by tertiles of protein intake using mean and standard deviation for continuous variables and percentages for categorical variables.

Linear regression was used to investigate the associations between substitution of dietary carbohydrate and (total, animal and plant) protein and 5-year weight (and waist) change. Cox regression was used to explore the association between these substitutions and all-cause and CVD mortality. For all regression analyses sex, age, energy-intake, BMI at recruitment, duration of diabetes, insulin use, education (four categories), physical activity index (four categories), smoking status at recruitment (three categories), and country were considered as confounding factors (model 1). For the linear regression for weight and waist change analyses, length of follow-up was included. Next, all analyses were adjusted for healthy diet by including vitamin C and fiber in the model (model 2). For subjects with missing values on physical activity index (n = 342), smoking status (n = 21), smoking intensity (n = 106), duration of diabetes (n = 410) or education (n = 273), values were imputed with multiple imputation in which 5 duplicate datasets were sampled, with the missing values replaced by imputed values. All confounding variables were used for the imputation, with the mortality (event) status, annual weight change, length of follow-up and dietary carbohydrates as role indicators. The results of these imputations were pooled with Rubin’s rules [31]. The proportional hazards assumption was checked for the Cox regression by log minus log plots (per quartile protein intake) with no deviations detected.

For energy-adjustment, we used the nutrient residual method (energy-adjusted) and performed sensitivity analyses using the multivariate nutrient density method [32]. In the nutrient residual method, the residuals from the regression of absolute intake of total protein, fat and alcohol intake (all per 10 gram) on total energy intake were obtained and rescaled by adding the mean population energy intake in the regression equations. Since total energy intake is an important predictor of the outcomes, total energy intake was also included in the model. Subsequently, the rescaled residuals were divided by 10 to generate intakes per 10 g/d. In the nutrient density method, the nutrient densities from total protein, fat, alcohol and total energy (per 5 energy%) were included as a covariate. Next, for both energy-adjustment methods total protein was additionally divided into animal and plant protein.

Interactions with 1) sex, 2) age, 3) BMI, 4) smoking status, 5) smoking intensity, 6) physical activity index and 7) GI were tested in the weight change and mortality sample and adjusted for all former listed possible confounders.

Sensitivity analyses were performed by excluding over- and under reporters on dietary intake and centers with high percentages of underreporters, and participants with prevalent chronic diseases (cancer, cardiovascular disease, stroke and cancer) at recruitment, and by adjusting for smoking intensity with eight categories and for alcohol intake (categories), all using the nutrient residual energy adjustment method. Alcohol consumption was divided into 7 categories with a daily consumption of 0 gram; >0-6 g; >6-12 g; >12-24 g; >24-60 g; >60-96 g; and >96 gram with >0-6 gram as reference category.

Finally, we used a meta-analytic approach to investigate heterogeneity across countries (STATA 11 metan procedure) by pooling the multivariate-adjusted HRs per country using the DerSimonian and Laird random effects model and testing for heterogeneity using a chi-square test.

Analyses were performed using the SPSS 20.0 statistic software package and P-values <0.05 were considered significant.

## Results and discussion

### Results

#### Baseline characteristics

Compared to participants in the highest tertile of protein intake, people in the lowest tertile were more likely to be male, higher educated, and had a lower BMI; they were less likely insulin users, current smokers and physically active and consumed more dietary carbohydrates, alcohol and energy intake. Age and duration of type 2 diabetes did not differ over the tertiles of protein intake. The mean energy intake was 2089 kcal (1805 kcal for females; 2321 kcal for males) with on average 42.3 (SD 7.2) en% carbohydrates, 18.2 (SD 3.3) en% total protein, 11.5 (SD 3.6) en% animal and 5.1 (SD 1.2) en% plant protein and 34.7 (SD 6.1) en% fat (Table 1). The baseline characteristics by tertiles of protein intake for the mortality sample were comparable (Table 2).

**Table 1:** **Characteristics of the population according percentage of energy from protein for weight change analysis**

	**Total**	**Tertiles of percentage of energy from protein**		
		**1**	**2**	**3**
**N**	**4082**	**1360**	**1361**	**1361**
Sex: male %(n)*	55.2% (2255)	63.1% (858)	55.1% (750)	47.5% (647)
Age [y]	57.5 (6.4)	57.5 (6.7)	57.6 (6.4)	57.3 (6.2)
	Male/female	Male/female	Male/female	Male/female
BMI [kg/m^2^]	28.4 (4.1)/29.3 (5.4)	28.3 (3.9)/28.8 (5.3)	28.4 (4.1)/29.2 (5.5)	28.6 (4.2)/29.6 (5.4)
Waist circumference [cm]	100.4 (11.1)/92.1 (13.1)	100.3 (10.7)/90.7 (12.8)	100.5 (11.2)/91.7 (13.2)	100.6 (11.3)/93.3 (13.2)
Duration of diabetes [y]^†^	4.4 (1.8-9.6)	4.3 (1.9-10.1)	4.7 (2.0-10.0)	4.1 (1.8-8.7)
Insulin use %(n)	20.9% (855)	19.8% (259)	22.1% (301)	21.7% (295)
Education				
Lower education	72.2% (2946)	71.6% (974)	71.4% (972)	74.8% (1018)
Physical activity index %(n)				
Active	39.7% (1621)	37.6% (511)	40.4% (549)	41.2% (561)
Smoking status %(n)				
Current smoker	24.5% (996)	22.5% (305)	24.9% (338)	26.0% (353)
Protein [energy%]	18.2 (3.3)	14.7 (1.5)	18.0 (0.8)	21.9 (2.0)
Animal	11.5 (3.6)	8.1 (1.9)	11.2 (1.7)	15.2 (2.6)
Plant	5.1 (1.2)	4.7 (1.1)	5.2 (1.2)	5.4 (1.2)
Protein [g]	94.2 (30.6)	81.0 (26.0)	94.6 (27.9)	106.9 (32.0)
Animal	59.6 (25.2)	45.1 (18.9)	59.4 (21.7)	74.3 (25.6)
Plant	26.1 (9.1)	25.3 (8.5)	26.9 (9.3)	26.3 (9.3)
Carbohydrates [energy%]	42.3 (7.2)	44.1 (7.9)	42.3 (6.7)	40.3 (6.5)
Fat [energy%]	34.7 (6.1)	34.2 (6.9)	35.0 (5.6)	34.8 (5.7)
Saturated [energy%]	13.0 (3.3)	13.5 (3.6)	13.1 (3.2)	12.5 (3.1)
Monounsaturated [energy%]	12.7 (3.3)	12.1 (2.9)	12.9 (3.1)	13.1 (3.8)
Polyunsaturated [energy%]	6.1 (2.1)	6.0 (2.2)	6.1 (1.9)	6.2 (2.3)
Alcohol [energy%]^†^	2.4 (0.3-7.3)	3.6 (0.9-11.2)	2.6 (0.4-7.1)	1.3 (0.1-4.7)
Total energy [kcal]	2089 (633)	2208 (684)	2098 (613)	1961 (575)

*Mean (SD), all such values; ^†^median (interquartiles), all such values.

Lower education is primary education or technical school; Active is moderately active or active.

410 duration of diabetes; 273 education, 21 smoking status 342 physical activity index were imputed.

**Table 2:** **Characteristics of the population according to percentage of energy from protein for mortality analysis**

	**Total**	**Tertiles of percentage of energy from protein**		
		**1**	**2**	**3**
**N**	**6107**	**2035**	**2036**	**2036**
Protein [energy%]*	18.0 (3.3)	14.6 (1.5)	17.9 (0.8)	21.7 (2.1)
Animal	11.3 (3.6)	8.0 (1.9)	10.9 (1.7)	15.0 (2.7)
Plant	5.1 (1.2)	4.8 (1.2)	5.2 (1.2)	5.3 (1.2)
Protein [g]	92.5 (30.4)	79.8 (26.0)	92.5 (27.8)	105.4 (31.6)
Animal	58.1 (24.9)	44.0 (18.5)	57.4 (21.2)	73.1 (25.5)
Plant	26.1 (9.3)	25.5 (9.2)	26.7 (9.5)	25.9 (9.1)
	Male/female	Male/female	Male/female	Male/female
Protein [g]	100.4 (31.9)/83.2(25.5)	85.9 (26.8)/68.8(20.4)	102.1 (28.6)/82.3(22.9)	118.1 (32.2)/93.7(26.1)
Animal	63.1 (26.5)/52.2(21.4)	48.1 (19.3)/36.8(14.3)	64.3 (22.1)/49.9(17.2)	82.0 (26.6)/65.0(21.4)
Plant	28.4 (9.9)/23.4(7.8)	27.1 (9.6)/22.7(7.7)	29.1 (10.0)/24.2(8.2)	29.1 (9.8)/22.9(7.3)
Carbohydrates [energy%]	42.7 (7.3)	44.8 (8.0)	43.0 (6.7)	40.4 (6.5)
Fat [energy%]	34.7 (6.2)	34.0 (7.0)	35.0 (5.6)	35.1 (5.7)
Saturated [energy%]	13.1 (3.3)	13.5 (3.7)	13.1 (3.1)	12.8 (3.1)
Monounsaturated [energy%]	12.7 (3.4)	12.1 (3.2)	13.0 (3.2)	13.1 (3.7)
Polyunsaturated [energy%]	6.0 (2.1)	5.8 (2.1)	6.0 (1.9)	6.1 (2.2)
Alcohol [energy%]^†^	2.1 (0.2-6.8)	3.2 (0.7-10.5)	2.1 (0.3-6.5)	1.1 (0.04-4.5)
Total energy [kcal]	2074 (639)	2196 (698)	2073 (620)	1953 (574)
Sex: male %(n)	54.5% (3328)	64.0% (1302)	51.7% (1052)	47.8 (974)
Age [y]	57.4 (6.7)	57.4 (6.7)	57.7 (6.4)	57.5 (6.3)
BMI [kg/m^2^]	28.8 (4.9)	28.4 (4.6)	28.9 (4.9)	29.3 (5.1)
Waist circumference [cm]	96.8 (13.0)	97.1 (12.7)	96.5 (12.9)	97.2 (13.3)
Duration of diabetes [y]^†^	4.3 (1.8-9.3)	4.1 (1.7-9.7)	4.8 (1.9-9.9)	4.2 (1.8-8.4)
Insulin use %(n)	21.3% (1302)	18.8% (382)	22.6% (460)	22.6% (460)
Education %(n)				
Lower education	71.6% (4372)	70.0% (1424)	70.4% (1433)	74.4% (1514)
Physical activity index %(n)				
Active	37.2% (2269)	35.6% (724)	36.5% (742)	39.4% (802)
Tobacco status %(n)				
Current smoker	25.0% (1522)	23.6% (478)	24.9% (504)	26.6% (540)

*Mean (SD), all such values; ^†^median (interquartiles), all such values; Lower education is primary education or lower vocational education; Active is moderately active or active; 410 duration of diabetes; 273 education, 21 smoking status 342 physical activity index were imputed.

### Weight and waist circumference change

In the weight-change sample the mean weight was 75.7 kg (SD 14.7) for females and 85.6 kg (SD 13.5) for males with an average 5-year weight change of −0.5 kg/y (SD 6.1) for females and −1.1 kg/y (SD 6.4) for males. In the waist change sample the average waist circumference was 92.1 (SD 13.1) cm for females and 100.4 (SD 11.1) cm for males with an average waist circumference change of 4.5 cm/5y (SD 6.4) for females and 2.2 cm/5y (SD 5.8) for males (Table 1).

Substituting dietary carbohydrates with total protein (β 187 [75;299] g/5y), and animal protein (β 196 [137;254] g/5y) showed a significant association with a higher 5-year weight change using both energy-adjustment methods after adjustment for vitamin C and fiber intake (model 2). Substitution with plant protein was only significant in the multivariate nutrient density model (model 1) (β 1080 [42;2117] g/5y), but lost significance after further adjustment for vitamin C and fiber intake. Additional adjustment for alcohol in categories showed similar results to model 2. No associations were found between substitution of carbohydrate with any protein and waist circumference change (Table 3).

**Table 3:** **Beta [95% CI] of 5-year weight and waist change for intakes of protein**

**Energy adjustment models**	**5-year weight change [g/5y]**		**5-year waist change [cm/5y]**	
*Nutrient residual model*	Model 1*	*Model 2* ^†^	Model 1*	*Model 2* ^†^
Total protein (10 g)	209 [96;316]	187 [75;299]	−0.03 [−0.20;0.14]	−0.04 [−0.22;0.13]
Animal protein (10 g)	203 [145;261]	196 [137;254]	−0.02 [−0.20;0.15]	−0.02 [−0.20;0.15]
Plant protein (10 g)	342 [−32;717]	82 [−421;584]	−0.02 [−0.64;0.60]	−0.77 [−1.51;0.04]
*Multivariate nutrient density model*				
Total protein (5 en%)	498 [199;796]	459 [157;761]	−0.15 [−0.64;0.33]	−0.18 [−0.67;0.31]
Animal protein (5 en%)	476 [170;783]	464 [156;771]	−0.15 [−0.41;0.34]	−0.15 [−0.64;0.35]
Plant protein (5 en%)	1080 [42;2117]	650 [−660;1961]	0.11 [−1.64;1.87]	−1.44 [−2.61;0.28]

N = 4,082 for 5-year weight change (2255 males and 1827 females); N = 1,898 for 5-year waist change. Two models were used for energy adjustment.

*Model 1: Beta, adjusted for protein intake (per 10 g / 5 energy%), alcohol intake (per 10 gram / 5 energy%), age at recruitment, BMI, duration of diabetes, insulin use (no/yes), education level (four categories), physical activity index (four categories), smoking status (three categories), sex, follow-up time and country. First total protein was added to the model. In the analysis of protein subtypes, mutual adjustments were made for animal and plant protein.

^†^Model 2: is model 1 with additional adjustments for healthy diet by including vitamin C and fiber in the model.

P < 0.05.

### All-cause and CVD mortality

After a mean follow-up period of 9.2 y (SD 2.3y), 787 (13%) participants had died, of whom 266 (4%) due to CVD in the (CVD) mortality sample. Substituting carbohydrates by plant protein was associated with a lower risk of all-cause mortality (HR 0.79 [0.64; 0.97]), but CVD mortality lost significance after adjustment for vit C and fiber (HR 1.03 [0.72;1.47]). Additional adjustment for alcohol in categories showed similar results to model 2. Substitution with animal and total protein was not associated with CVD or all-cause mortality with both energy adjustment methods (Table 4).

**Table 4:** **Hazard ratio [95% CI] of all-cause and cardiovascular mortality for intakes of protein**

**Energy adjustment models**	**HR**			
	**All-cause mortality**		**CVD mortality**	
*Nutrient residual model*	Model 1*	*Model 2* ^†^	Model 1*	*Model 2* ^†^
Total Protein (10 g)	0.96 [0.92;1.01]	0.99 [0.94;1.03]	0.95 [0.88;1.03]	1.00 [0.92;1.08]
Animal Protein (10 g)	0.99 [0.95;1.04]	1.00 [0.95;1.04]	0.99 [0.92;1.07]	1.00 [0.93;1.09]
Plant Protein (10 g)	0.71 [0.61;0.82]	0.79 [0.64;0.97]	0.69 [0.54;0.90]	1.03 [0.72;1.47]
*Multivariate nutrient density model*				
Total Protein (5 en%)	0.94 [0.84;1.06]	1.00 [0.88;1.12]	0.91 [0.74;1.11]	1.00 [0.82;1.23]
Animal Protein (5 en%)	1.00 [0.89;1.12]	1.01 [0.90;1.14]	0.98 [0.80;1.20]	1.02 [0.83;1.25]
Plant Protein (5 en%)	0.39 [0.26;0.57]	0.55 [0.32;0.93]	0.31 [0.16;0.62]	0.81 [0.33;1.99]

N = 6,107 with 787 cases all-cause mortality and 266 cases in cardiovascular mortality.

Two models were used for energy adjustment.

*Model 1, Hazard ratio (HR) respectively Beta, adjusted for energy intake, protein intake (per 10 g / 5 energy%), alcohol intake (per 10 gram / 5 energy%), age at recruitment, BMI, duration of diabetes, insulin use (no/yes), education level (four categories), physical activity index (four categories), smoking status (three categories), sex, and country.

^†^Model 2, is model 1 with additional adjustments for healthy diet by including vitamin C and fiber in the model.

P < 0.05.

### Interactions and sensitivity analyses

Only the interaction between GI and total protein was significant (p = 0.04) in the mortality sample (p < 0.0001), but not for CVD mortality (p = 0.14) or in the weight change sample (p = 0.30). In the high GI stratum, substitution of carbohydrates with total protein was associated with a lower all-cause mortality risk (0.93 (0.87;0.99)) than the low GI stratum (1.04 (0.97;1.10)).

After excluding energy over- and under-reporters (n = 1446 excluded; β 164 [44; 283] g), or people with chronic illness at recruitment (N = 426 excluded; β 203 [83; 323] g) showed similar results for the association between substitution of carbohydrate with protein and weight change in model 2. The energy under-reporters were mainly participants from one German and one Dutch center. Leaving out participants of this German center or of both centers attenuated the associations with weight change to borderline significant for total protein intake, while the association for animal protein intake remained significant. Additional adjustment for alcohol in categories did not change the results. When pooling estimates for different countries for associations with weight change or all cause mortality, no significant heterogeneity was observed (I^2^ < 52%).

## Discussion

In this prospective study among European patients with type 2 diabetes, we found that isocaloric substitution of carbohydrates with total, animal or plant protein was associated with a higher 5-year weight gain. However, substitution with plant protein lost significance after adjustment for a healthy diet. Substitution of carbohydrate with any type of protein was not associated with waist circumference change. Substitution with plant protein was associated with lower all-cause mortality risk.

### Weight gain

The substitution of dietary carbohydrate with protein was associated with weight gain, but not with waist circumference change. In patients with type 2 diabetes, two short term randomized trials with both 30% protein and 40% carbohydrate versus 15% protein and 55% carbohydrate found no significant difference in weight change after one year [15,16], whereas another study found significantly higher total and abdominal fat in women on a high protein diet; but not in men [17]. We are not aware of such long-term studies in patients with type 2 diabetes. In the general population, studies under iso-energetic conditions found no significant difference on weight change between high-protein or high-carbohydrate diets in the short term [8,11]. In observational studies, recently one study showed that a higher protein intake was associated with weight gain [10]. Another study showed that substitution of carbohydrate with protein was not associated with weight gain [33]. Evidence in the long term is thus inconclusive [12,13]. Our study showed that substitution of dietary carbohydrates with total protein was associated with weight gain. This weight gain was relatively small, ranging from 187 to 196 g/y and the association with substitution for plant protein lost significance after adjustment for a healthy diet. Whereas the total EPIC population gained weight, the patients with type 2 diabetes lost weight while their waist circumference increased. This difference could be caused by under-reporting of self-reported follow-up weight, but it is hard to verify this. Alternatively, this could be intentional weight loss [34]. Finally, it is well established that diabetes patients tend to be more katabolic with loss of lean tissue [35]. Possibly, the diabetes patients in our study had a concomitant loss of lean tissue mass and gain of fat mass. The higher protein intake might have prevented loss of lean body mass.

In the general population, one study suggested differences in effects of animal and plant protein on weight, with a direct association of animal protein and risk of overweight and obesity, and an inverse association for plant protein [14]. Our study confirmed that substitution of carbohydrate with total and animal protein was associated with a higher 5-year weight. However, the inverse association for plant protein on weight change could not be confirmed in patients with type 2 diabetes. This might at least partially be caused by lower mean intakes of plant protein (mean intake 26 (SD 9) g/day) than animal protein (mean intake 60 (SD 25) g/day).

### Mortality risk

In our study, we found that substituting of carbohydrate with plant protein was associated with lower (CVD) mortality. This confirms the inverse association between plant protein and CVD mortality in the general population [12], although we did not observe a higher (CVD) mortality risk in substitution of carbohydrates with total protein or animal protein. After adjustment for healthy diet, the association with CVD mortality lost significance, but the association with lower risk for all-cause mortality remained significant, probably due to the higher number of all-cause mortality cases. This suggests that a healthy diet only partially explains the association between substitutions of carbohydrates with plant protein. Furthermore, since plant protein is more often associated with higher fiber and vitamin C intakes, this adjustment for a healthy diet with vitamin C and fiber probably led to over-adjustment. Such diets high in fruit and vegetables have cardioprotective effects through several nutrients such as vitamin C, fiber or carotenoids [36,37]. Since these nutrients are likely intermediates in the causal pathway, adjustment for such factors would be over-adjustment and lead to underestimation of the association.

### Type of carbohydrates

In our analyses we studied the association between substitutions of total carbohydrates. However, the type of carbohydrate that is replaced also plays a role, since GI of the diet has been associated with CVD risk [38]. We therefore investigated the interaction of GI of the diet with carbohydrate substitution. We found a significant interaction between total protein and GI of the diet for all cause mortality. In the high GI stratum, substitution of carbohydrates with protein was associated with lower all-cause mortality risk than in the low GI stratum. Therefore, substituting carbohydrates from the high GI stratum seems to be more beneficial. This is in line with a recent meta-analysis which suggesting that GI might play a role especially in diabetes patients [38]. However, a previous study in this population found no association between GI and mortality risk [39].

### Strengths and limitations

Strengths of the study are its prospective design, which limits the potential for reverse causation, the large number of participants, inclusion of both men and women, and the use of participants from 15 cohorts across European countries followed for nine years with widely varying dietary intakes [18]. In an attempt to minimize the effect of confounding from clustering of healthier lifestyles that might be associated with higher intakes of dietary protein, our analyses were adjusted for a comprehensive range of potential confounders, including dietary, lifestyle and socioeconomic factors.

There are some study limitations. A FFQ for dietary assessment was only used at recruitment. We did not examine any changes in intake during follow-up, which might vary over time. However, excluding participants most likely to have changed their diets (those with chronic disease at recruitment) did not alter our findings. Furthermore, assessment of the long-term reproducibility of the FFQ in the EPIC-Heidelberg cohort showed fairly high correlation between measurements at recruitment and at follow-up (correlation coefficients: 0.41 – 0.77) [40]. In the Northern cohort, however, the fat intake decreased between 1986 and 1992, followed by an increase after 2004 with the introduction of the low carbohydrate-high-fat diet. This might have altered the CVD risk in this particular region [41]. The use of self-reported dietary questionnaires potentially resulted in the underreporting of fat intake [42], but the use of energy-adjusted methods minimizes such potential misclassification [32]. In addition, exclusion of subjects, who underreported dietary intake according to the Goldberg criterion, did not alter our findings [43]. Finally, weight and waist circumferences at follow-up were self-reported, which may lead to potential underestimation from self-report, particularly with regard to the waist circumference. However, results in the two other centers within the EPIC cohort with measured weight and waist circumferences were in agreement with the rest of the cohort [13].

## Conclusions

To conclude, isocaloric substitution of carbohydrates with total and animal protein may increase body weight in patients with type 2 diabetes. Substitution of carbohydrates with plant protein may reduce the risk of total and probably CVD mortality. Therefore, future study is needed whether dietary guidelines should not actively promote replacement of carbohydrates by total protein, but rather focus on replacement of carbohydrates with plant protein.

## Abbreviations

BMR: Basal metabolic rate
CVD: Cardiovascular disease
EPIC: European Prospective Investigation into Cancer and Nutrition
FFQ: Food frequency questionnaires
GI: Glycemic index
